# Serum Leukocyte Immunoglobulin-Like Receptor A3 (LILRA3) Is Increased in Patients with Multiple Sclerosis and Is a Strong Independent Indicator of Disease Severity; 6.7kbp LILRA3 Gene Deletion Is Not Associated with Diseases Susceptibility

**DOI:** 10.1371/journal.pone.0149200

**Published:** 2016-02-12

**Authors:** Hongyan An, Chai Lim, Gilles J. Guillemin, Ute Vollmer-Conna, William Rawlinson, Katherine Bryant, Nicodemus Tedla

**Affiliations:** 1 Inflammation and Infection Research Centre, School of Medical Sciences, Department of Pathology, University of New South Wales, Sydney, NSW 2052, Australia; 2 MND and Neurodegenerative diseases Research Centre, Australian School of Advanced Medicine (ASAM), Macquarie University, Sydney, NSW 2109, Australia; 3 School of Psychiatry, University of New South Wales, Sydney, NSW 2050, Australia; 4 Department of Virology, South Eastern Sydney and Illawarra Health Service, Prince of Wales Hospital, Sydney, NSW 2032, Australia; 5 South Western Sydney Clinical School, University of New South Wales, Sydney, NSW 2170, Australia; Universite Lyon 1, FRANCE

## Abstract

Leukocyte immunoglobulin-like receptor A3 (LILRA3) is a soluble immune regulatory molecule primarily expressed by monocytes and macrophages. A homozygous 6.7kbp LILRA3 gene deletion that removes the first seven of its eight exons is predicted to lead to lack of LILRA3 protein, although this has not been experimentally confirmed. Moreover, there are conflicting results with regards to the link between the LILRA3 homozygous genetic deletion and susceptibility to multiple sclerosis (MS) in different European populations. The aim of this study was to investigate whether LILRA3 gene deletion is associated with MS susceptibility in a North American cohort of European ancestry and assess if serum LILRA3 protein level is a marker of clinical subtype and/or disease severity in MS. A total of 456 patients with MS and 99 unrelated healthy controls were genotyped for the 6.7kbp LILRA3 gene deletion and levels of LILRA3 protein in sera determined by in-house sandwich ELISA. We showed that LILRA3 gene deletion was not associated with MS susceptibility and did not affect the age of disease onset, clinical subtype or disease severity. However, we discovered for the first time that homozygous LILRA3 gene deletion results in lack of production of LILRA3 protein. Importantly, LILRA3 protein level was significantly increased in sera of patients with MS when compared with control subjects, particularly in more severe type primary progressive MS. Multiple regression analysis showed that LILRA3 level in serum was one of the strongest independent markers of disease severity in MS, which potentially can be used as a diagnostic marker.

## Introduction

Multiple sclerosis (MS) is a complex autoimmune disorder directed against components of CNS myelin or oligodendrocytes (OGD), probably initiated by environmental factors such as infections in genetically susceptible individuals [1–5]. About 85% of patients initially present with relapsing remitting disease (RRMS), which is characterised by recurrent and reversible neurological deficits [6, 7]. With time, the majority of these patients will progress to the secondary progressive phase (SPMS) with continuous irreversible neurological decline [6, 7]. 15% of patients are diagnosed with primary progressive MS (PPMS) and show severe progression of disability with no remission phase(s) [6, 7]. Progressive relapsing MS (PRMS) is a rare clinical pattern (<5% of patients) characterised by several recurrent attacks from onset with little or no improvement [6]. Factors regulating clinical variability and/or disease severity are not fully elucidated. However, variants in the Human Leukocyte Antigen (HLA) genes from the Major Histocompatibility Complex (MHC) in chromosome 6p21 have been consistently linked with MS susceptibility (reviewed in [4]). In some studies chromosome 19q13 has been found to be linked to MS [8, 9] and recent genome wide association studies have identified 110 MS risk variants in 103 discrete loci outside of the Major Histocompatibility Complex [4, 10–14].

LILRA3 is a soluble molecule that belongs to a family of highly homologous activating and inhibitory cell surface receptors [15], primarily expressed by mono-myeloid cells [16, 17]. LILRs are increasingly recognized as critical regulators of innate immune responses through modulation of the threshold and amplitude of leukocyte activation [16–19]. Functions of the soluble LILRA3 are not fully elucidated; however, its close sequence similarity to the extracellular domains of activating LILRA1 and LILRA2 and inhibitory LILRB1 [16, 20], suggests that it may act as a soluble antagonist/agonist to these receptors via shared ligands. Interestingly, LILRA3 located in chromosome 19q13.4, is the only LILR showing genetic diversity, with one or two LILRA3 allelic deletions of 6.7kbp removing the first seven of its eight exons [21]. This deletion is found in different populations worldwide at different rates. The deletion occurs at extremely high frequency in Northeast Asians such as Japanese (71%), Chinese (79%) and Korean (84%) compared to European (15–25%), Middle Eastern (10%) or African (7%) populations [21–27]. The occurrence of homozygous LILRA3 gene deletion “null allele” that predicts loss of gene expression in these populations ranges from 1.6% to 45% [21, 23].

There are several reports linking LILRA3 deletion polymorphism to various autoimmune diseases (reviewed in [28]). Of particular interest here are the conflicting results with regards to the link between homozygous LILRA3 gene deletion and the susceptibility to MS. Lack of LILRA3 gene has been reported to be a risk variant in German [29] and Spanish populations [24] but not in Polish [25] and Finnish population [8], despite all having comparable frequencies of LILRA3 gene deletion in their general populations. In this study we aim to investigate whether LILRA3 gene deletion is linked with MS susceptibility in a North American cohort; additionally we will for the first time i) assess whether LILRA3 null allele leads to lack of LILRA3 protein expression as predicted, ii) determine LILRA3 protein levels in patients with MS and healthy controls, and iii) investigate if LILRA3 protein levels correlate with clinical subtype, and/or disease severity in MS.

## Materials and Methods

### Study cohort

Archival sera from 456 patients with MS manifesting different clinical patterns of the disease and 99 age sex and race-matched unrelated healthy controls were obtained from The Accelerated Cure Project for MS, USA. The patient cohort included 272 patients with relapsing remitting (RRMS), 133 with secondary progressive (SPMS) and 51 with primary progressive (PPMS) disease. Two sets of sera (12 months apart) were available for 18 patients with RRMS; six of these patients had no change in disease severity, six had improvement and six had worsening disease as measured by Expanded Disability Status Scale (EDSS) disease scores. These sera were used to investigate whether LILRA3 protein levels fluctuated with changing disease severity. All healthy subjects and patients were North Americans of European descent. Diagnosis of MS was according to the McDonald classification [30], and all patients were on one or more clinically-approved treatment prior to and at the time of this study. This study was approved by institutional human ethics committees and informed consent was obtained from each subject.

### LILRA3 genotyping

**Fig 1:**
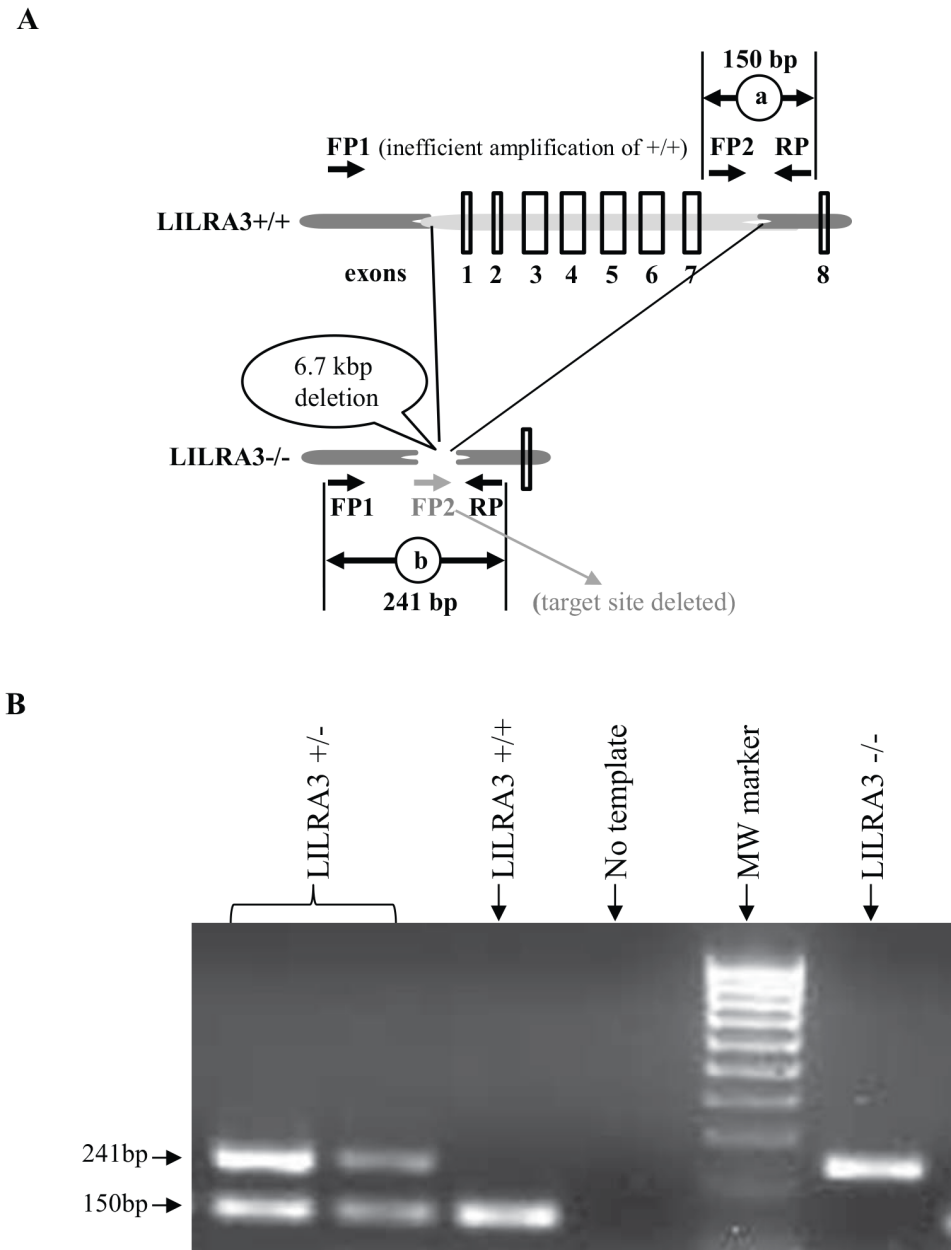
LILRA3 genotyping schematic diagram and representative results. **(A)** Schematic illustration of LILRA3 genotyping strategy used to distinguish LILRA3 gene deletion polymorphisms (adapted from [24]). Two forward (FP1 and FP2), and one reverse (RP) primers were used in a single PCR reaction. In subjects with two intact LILRA3 alleles, FP1 primer which is upstream from the 6.7kbp deletion together with the RP will poorly amplify the very large size product spanning the entire 8 LILRA3 exons; instead a single 150bp product will be efficiently amplified by the downstream FP2 primer. Subjects who are heterozygous will show a 241bp product from the deleted allele and a 150bp product from the intact allele, and subjects with homozygous gene deletion will preferentially amplify the 241bp product. **(B)** A representative 2% agarose gel for LILRA3 PCR products showing two patients with MS having heterozygous gene deletion (LILRA3+/-; lane 1, 2), one patient showing two intact LILRA3 alleles “wild type” (LILRA3+/+; lane 3) and one patient showing a homozygous deletion “null allele” (LILRA3 -/-; lane 6). Lane 4 is a no template control and lane 5 is the molecular weight marker.

Genomic DNA was extracted from 100 μl of serum of each subject using QIAamp DNA Blood mini kit (QIAGEN, VIC, Australia) according to the manufacture’s instruction. A PCR-based genotyping for wild type, heterozygous or homozygous LILRA3 gene deletion was performed as described [24]. The following three LILRA3 primers (Sigma-Aldrich, NSW, Australia) were used in a single PCR reaction: a forward primer upstream from the 6.7kbp deletion FP1 (5'- GAC TTG TAA GGG TTA AAA AGC CAA-3'), an internal forward primer within the deletion FP2 (5'- CAT CTC GAT CTG CCA CTG ACA C-3') and a reverse primer RP (5'- GAC AGC AGA TTC TAA AAC AGT GG-3') (Fig 1A). In brief, a 20 μl PCR reaction mix containing 100 ng of genomic DNA as template, 0.2 mM dNTP, 2.5 mM MgCl_2_, 0.3 μM of each primer and 0.5 μl AmpliTaq Gold® DNA polymerase (Life Technologies, VIC, Australia) was amplified using the following conditions: 95°C for 5 minutes initial annealing, 35 cycles of 95°C for 30 seconds annealing, 60°C for 45 seconds and 72°C for 45 seconds extension, and a final 10 minutes extension at 72°C. Reactions with no template were used as negative controls. The PCR products were separated by electrophoresis in 2% agarose gels and analysed using in gel doc XR systems (Bio-Rad, NSW, Australia). A single 150bp PCR product amplified by FP2 and RP is expected in subjects with intact LILRA3 gene in both alleles (LILRA3+/+) and a single 241bp PCR product amplified by FP1 and RP is expected in subjects with homozygous gene deletion (LILRA3-/-); subjects with a single gene deletion are expected to have both products (LILRA3+/-) (Fig 1B).

### LILRA3 Sandwich ELISA and Mutliplex Cytokine assays

LILRA3 sandwich ELISA modified from our previous direct ELISA [31] was developed in-house with improved sensitivity. In brief, flat-bottom 96-well Nunc Maxisorp plates (Thermo Fisher Scientific, VIC, Australia) were coated with 100 μl/well of 0.5 μg/ml anti-LILRA3 monoclonal Ab clone2E9 (M01; Abnova, Taipei, Taiwan) in PBS at room temperature (RT) overnight. Plates were then washed 3 times with 0.05% Tween 20 in PBS, pH 7.2 (wash buffer) and blocked with 1% BSA in PBS (blocking buffer) for 2 hours at RT. This was followed by incubating 100 μl/well of serially diluted recombinant LILRA3 standards and test sera (1:10 dilution in blocking buffer) at 4°C overnight. Next day after 3 times washes, plates were incubated with 100 μl/well of 0.5 μg/ml anti-LILRA3 purified MaxPab rabbit polyclonal Ab (D01P; Abnova, Taipei, Taiwan) in blocking buffer at 4°C overnight. After 4 stringent washes, plates were incubated with 100 μl/well of Horse radish peroxidase (HRP)-conjugated goat anti-rabbit Ab (1:2000 dilution; Bio-Rad, NSW, Australia) for 1.5 hours at RT following with 4 washes. Finally, 100 μl/well of 3,3',5,5'-Tetramethylbenzidine chromogenic HRP substrate (Thermo Fisher Scientific, VIC, Australia) was added and incubated for 30 minutes in the dark and reaction was stopped by adding 50 μl/well of 1N H_2_SO4. Optical density was measured at 450/540 nm wavelength using the Spectramax-M3 plate reader (Molecular Devices, CA, USA).

Bio-Plex multi-cytokine bead assay kit with detection limits between 2-5pg/ml was used to simultaneously measure levels of IL-10, IFNγ and TNFα in sera according to the manufacturer’s instructions (Bio-Plex^TM^ Cytokine Assay, Bio-Rad Laboratories, CA, USA).

### Statistical analysis

All analyses were performed in SPSS Statistics Software for Windows, Version 21.0 (IBM Corp., NY, USA) and GraphPad InStat software version 6.05 (CA, USA). Retrospective power analyses were performed for available controls and patients using a fixed minor allele frequency of 25%, a type I error p value of 0.05, and an odds ratio (OR) of 1.25.

Deviation of LILRA3 genotype counts from the Hardy-Weinberg equilibrium was tested using Pearson’s goodness-of fit chi-square test. Two-sided Fisher’s exact test was used to analyse differences in allelic frequencies, genotype distribution and phenotype distribution of the 6.7kbp LILRA3 deletion between patients with MS and control subjects.

Mann-Whitney test was used to compare serum LILRA3 levels in controls among the different LILRA3 genotypes and to compare levels between patients with MS and controls. The difference in LILRA3 protein among the different clinical subtypes of MS or EDSS was analysed by one way ANOVA with Dunn’s post-test for multiple comparisons. Spearman r was used to correlate serum LILRA3 level to EDSS or serum cytokine levels. Paired t test was used to assess change in LILRA3 levels in patients that improved, worsened or not changed their EDSS over 12 months. P value <0.05 was considered statistically significant.

Multiple regression analysis using the SPSS ENTER METHOD was employed to evaluate predictors of disease severity using LILRA3 protein levels, age of disease onset, sex, recent disease exacerbation and age at the time of blood collection as covariates. Collinearity statistics were very acceptable. The mean variance inflation factor (VIF) was 1.33 (range: 1.01–1.88); and mean tolerance values were 0.80 (range: 0.53–0.99). The model was highly significant [F (5,166) = 10.1; p<0.001] and predicted approximately 23% (R^2^) of the variance in the outcome variable.

## Results

### Demography of study cohort

As shown in Table 1, the median age of patients at the time of the blood collection was 48 ± 11.0 (median ± SD) years which was comparable to control subjects (46 ± 10.8 years). The female to male ratios of patients and control subjects were 3.1 and 3.3 respectively. The median age of disease onset for patients with primary progressive disease (PPMS) was 46 ± 9.8 years, which was older than patients with relapsing remitting disease (RRMS; 37 ± 10.2 years) or patients with secondary progressive disease (SPMS; 39 ± 10.3 years) but not statistically significant. Disease duration for all patients ranged from 3 months to 38 years and patients with SPMS had the longest disease duration (median 13 ± 8.7 years) when compared to all other clinical subtypes. The mean disease severity score according to the EDSS was 2.8 ± 1.8 for patients with RRMS, 5.3 ± 2.0 for SPMS and 4.7 ± 2.1 for PPMS, indicating that patients with SPMS and PPMS had more severe (active) disease at the time of blood collection when compared to patients with RRMS.

**Table 1:** Demography of study cohort.

	*Controls (n = 99)*	*All MS (n = 456)*	*RRMS (n = 272)*	*SPMS (n = 133)*	*PPMS (n = 51)*
***Female to Male ratio***	3.3:1	3.1:1	4.1:1	2.1:1	2.2:1
***Median age ± SD***	46 ± 10.8	48 ± 11.0	43 ± 10.6	53 ± 9.3	52 ± 9.9
***Median age of disease onset ± SD***	N/A	39 ± 10.5	37 ± 10.2	39 ± 10.3	46 ± 9.8
***Median disease duration ± SD (Yrs)***	N/A	6 ± 7.9	5 ± 6.7	13 ± 8.7	5 ± 5.2
Mean EDSS ± SD*	N/A	3.8 ± 2.1	2.8 ± 1.8	5.3 ± 2.0	4.7± 2.1

EDSS: Expanded Disability Status Scale, MS: multiple sclerosis, RRMS = relapsing remitting MS, SPMS = secondary progressive MS, PPMS = primary progressive MS

*EDSS at the time of blood collection was available from 192 patients with MS; RRMS (116), SPMS (52), PPMS (24)

### LILRA3 genotyping in patients with MS and healthy controls

The distribution of LILRA3 genotypes in control subjects and patients with MS was in conformity with Hardy Weinberg equilibrium (X^2^ = 0.11 and 0.18 respectively, p>0.05). Table 2 shows the allelic, genotypic and phenotypic frequencies of LILRA3 gene deletion polymorphisms in control subjects and patients with MS. The odd ratio of LILRA3 allelic frequencies and phenotypes for MS susceptibility were 1.35 (95% CI = 0.93–1.96; p = 0.131) and 2.42 (95% CI = 1.00–5.82; p = 0.055) respectively. The proportion of control subjects with homozygous LILRA3 gene deletion were higher (8.1%) than patients with MS (3.6%), and both groups had similar proportions of heterozygous deletion. There was no statistically significant difference in the distribution of LILRA3 gene deletion between controls and patients. Although not statistically significant, we noticed that homozygous LILRA3 gene deletion was higher in male controls and patients when compared to their corresponding female counterparts (Table 3). There was no significant association between LILRA3 gene deletion and MS susceptibility when analysed using sex as a covariant. Logistic regression analyses showed no significant association between LILRA3 deletion (-/- and -/+) and clinical subtype; RRMS (n = 272, p = 0.69), SPMS (n = 133, p = 0.28) or PPMS (n = 51, p = 0.33). Similarly, linear regression analyses revealed no significant link between LILRA3 gene deletion and age of disease onset (p = 0.80) or disease severity (p = 0.79).

**Table 2:** Distribution of allelic frequency, genotypes and phenotypes of LILRA3 deletion in control subjects and patients with multiple sclerosis.

*A*. *Allelic frequency*	*Controls (n = 99)*	*Patients (n = 456)*	*P value	*Odd ratio*	*95% CI*
***LILRA3+***	153 (77.3%)	749 (82.1%)	*0*.*131*	*1*.*35*	*0*.*93–1*.*96*
***LILRA3-***	45 (22.7%)	163 (17.9%)			
***B*. *Genotypic frequency***					
***LILRA3+/+***	62 (62.6%)	309 (67.7%)	*0*.*347*	*0*.*80*	*0*.*51–1*.*25*
***LILRA3+/-***	29 (29.3%)	131 (28.7%)	*0*.*903*	*1*.*03*	*0*.*64–1*.*66*
***LILRA3-/-***	8 (8.1%)	16 (3.6%)	*0*.*055*	*2*.*42*	*1*.*00–5*.*82*
***C*. *Phenotypic frequency***					
***LILRA3+***	91 (91.9%)	440 (96.4%)	*0*.*055*	*2*.*42*	*1*.*00–5*.*82*
***LILRA3-***	8 (8.1%)	16 (3.6%)			

*Fisher’s exact test

CI: Confidence interval

**Table 3:** Distribution of genotypes of LILRA3 deletion in female and male control subjects and patients with multiple sclerosis.

	*Controls (n = 99)*		*Patients (n = 456)*	
*Genotypic frequency *	*female (n = 76)*	*male (n = 23)*	*female (n = 344)*	*male (n = 112)*
***LILRA3+/+***	46 (60.5%)	16 (69.6%)	235 (68.3%)	74 (66.0%)
***LILRA3+/-***	24 (31.6%)	5 (21.7%)	99 (28.8%)	32 (28.6%)
***LILRA3-/-***	6 (7.9%)	2 (8.7%)	10 (2.9%)	6 (5.4%)

### Links between 6.7kb LILRA3 gene deletion and LILRA3 protein production

**Fig 2:**
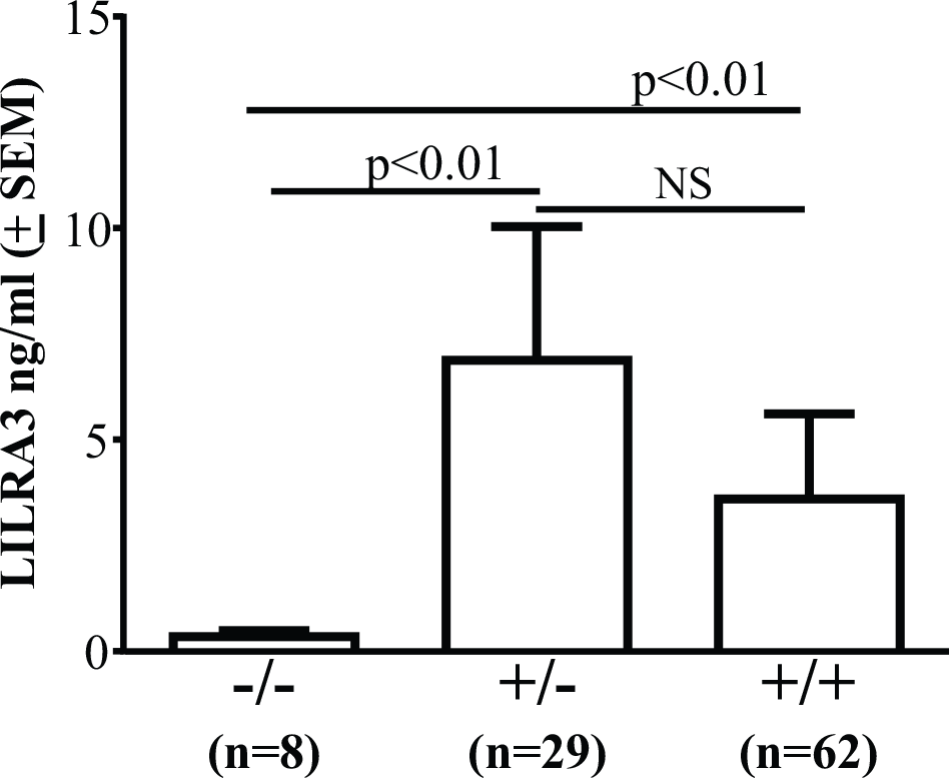
Links between LILRA3 genotype and protein expression. Detection of LILRA3 protein in sera of healthy subjects using an in-house sandwich ELISA showing no or little protein in subjects with LILRA3 gene null alleles (-/-) as contrasted to constitutive expression in subjects with heterozygous gene deletion (+/-) or wild type gene (+/+).

The 6.7kbp LILRA3 gene deletion is expected to cause lack of LILRA3 protein as predicted by the putative loss of the coding region but this was not experimentally confirmed. To address this, LILRA3 in sera of all healthy controls was determined and levels compared to their matching LILRA3 genotype. All eight subjects with homozygous gene deletion (LILRA3-/-) had very little detectable LILRA3 protein (0.36 ± 0.1 ng/ml) (Fig 2). In contrast, abundant protein was detected in sera of subjects with wild type LILRA3 (+/+; n = 62) and heterozygous LILRA3 gene deletion (+/-; n = 29) (Fig 2). Surprisingly, mean LILRA3 protein levels in subjects with LILRA3+/- genotype was higher (6.9 ± 3.2 ng/ml) than individuals with wild type LILRA3+/+ genotype (3.6 ± 2.0 ng/ml), although this was not statistically significant.

### Comparisons of LILRA3 protein levels in sera of patients with MS and healthy controls

**Fig 3:**
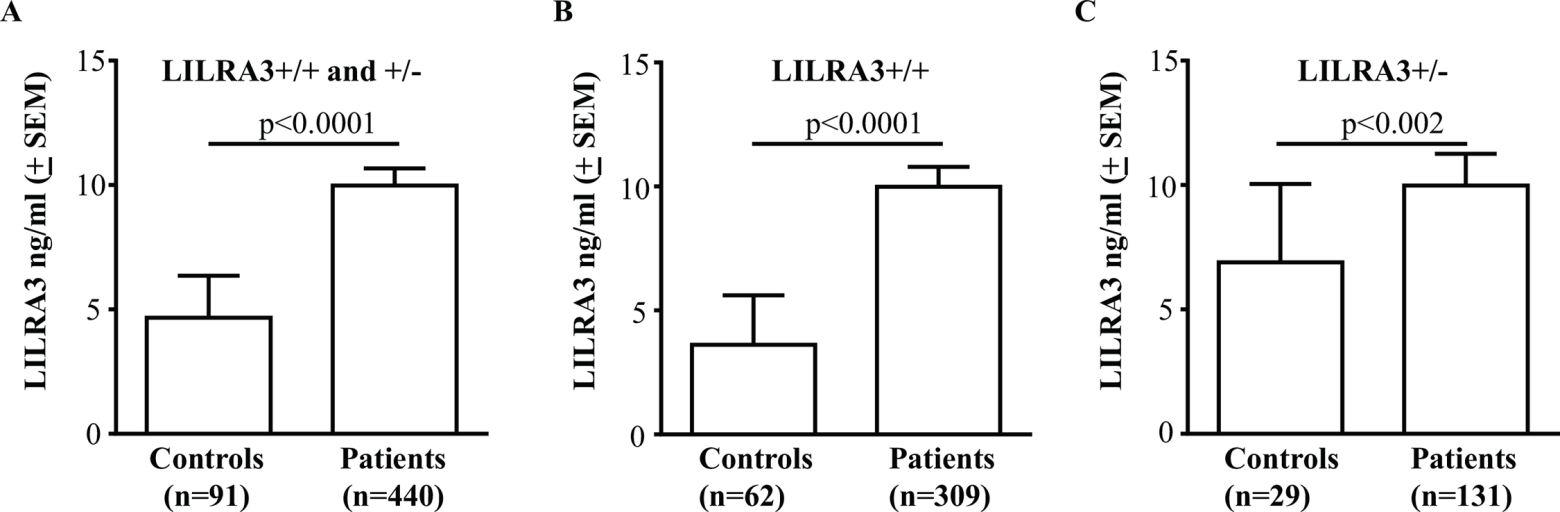
Comparison of serum LILRA3 level between patients with MS and healthy controls. **(A)** Serum LILRA3 level was significantly higher in patients with MS as compared to healthy control subjects. This significant difference was consistent when comparing levels in patients and control subjects with wild type (+/+) **(B)** or heterozygous LILRA3 gene deletion (+/-) **(C)**. Data are presented as mean ± SEM and analysed using Mann Whitney test.

**Fig 4:**
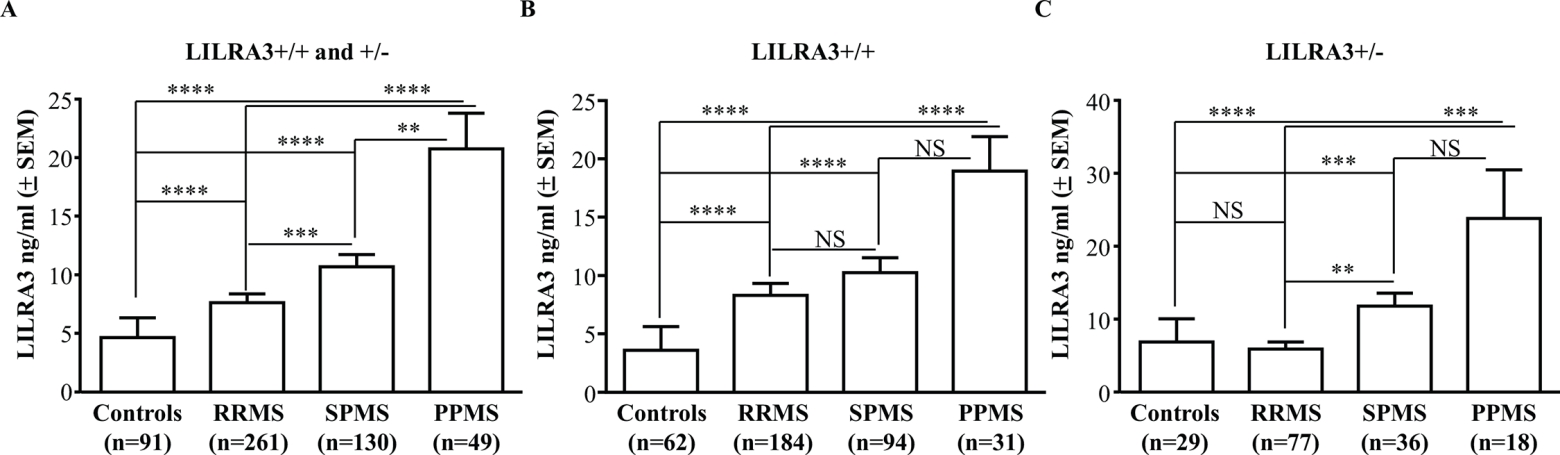
Comparison of serum LILRA3 level among different subtypes of MS. **(A)** Expression of LILRA3 in sera of patients with different clinical subtypes of MS showing progressive and significant increase in the amount of LILRA3 in sera of patients with RRMS, SPMS and PPMS when compared to control subjects. Similar results were obtained when patients with different clinical subtypes and the control subjects were stratified based on their LILRA3 genotype to wild type **(B)** and heterozygous deleted subgroups **(C)**. Data are presented as mean ± SEM and analysed using one way ANOVA with Dunn’s post-test for multiple comparisons (**p<0.01, ***p<0.001, ****p<0.0001).

LILRA3 levels in sera of patients with MS were compared with sex, age, and race-matched healthy subjects. The small number of controls (n = 8; 8.1%) and patients (n = 16; 3%) with homozygous LILRA3 gene deletion was excluded. The average quantities of LILRA3 in sera of patients with MS was 9.98 ± 0.7 ng/ml (n = 440) that was significantly higher than the levels in healthy controls (4.7 ± 1.7 ng/ml; n = 91) (p<0.0001; Fig 3A). Stratification based on LILRA3 genotype confirmed similar significant increase in LILRA3 protein in patients as compared to the corresponding controls for both LILRA3+/+ (9.97 ± 0.8 ng/ml versus 3.6 ± 2.0 ng/ml; p<0.0001; Fig 3B) and LILRA3+/- (9.9 ± 1.3 ng/ml versus 6.9 ± 3.2 ng/ml; p<0.002; Fig 3C) subjects. Analysis of serum LILRA3 in patients with the different clinical subtypes showed that patients with primary progressive disease (PPMS) showed ~3 times more LILRA3 (20.7 ± 3.1 ng/ml; n = 49) when compared to patients with relapsing remitting disease (RRMS) (7.6 ± 0.8 ng/ml; n = 261) and had ~2 times more than patients with secondary progressive disease (SPMS) (10.7 ± 1.1 ng/ml; n = 130) (Fig 4A). LILRA3 levels in PPMS were significantly more compared to RRMS (p<0.0001) and SPMS (p<0.05) (Fig 4A). These results were consistent when patients were further stratified on the basis of their LILRA3+/+ (Fig 4B) or LILRA3+/- (Fig 4C) genotype, although some did not reach statistical significance.

### Relationships between serum LILRA3 levels and EDSS in MS

**Fig 5:**
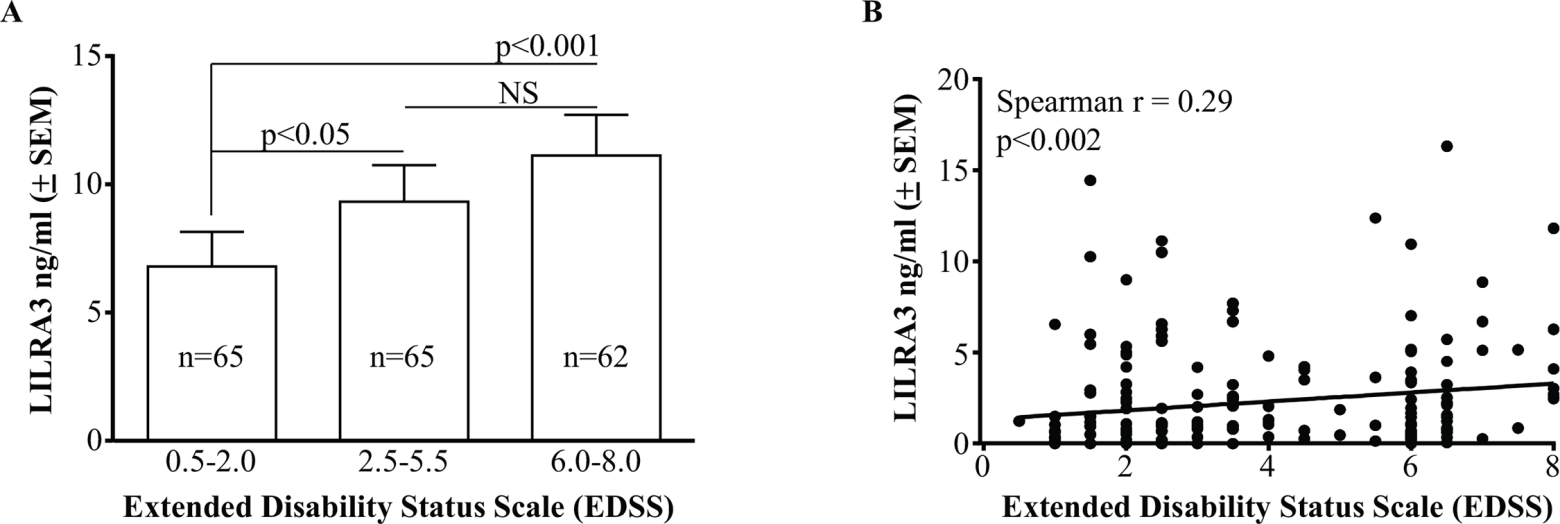
Association of serum LILRA3 with disease severity (EDSS). **(A)** Patients with MS in remission (EDSS scores 0.5–2) had significantly lower levels of serum LILRA3 when compared to patients with moderate disease severity (EDSS 2.5–5.5) and patients with severe disease (EDSS 6.0–8.0); Data are presented as mean ± SEM and analysed using one way ANOVA with Dunn’s post-test for multiple comparisons. **(B)** Spearman analysis confirming significant positive correlation between increasing levels of LILRA3 in serum and EDSS scores in patients with MS (r = 0.29, p<0.002).

The possible impact of LILRA3 levels on disease severity (reflected in EDSS scores at the time of sera collection) was determined in LILRA3+/+ and +/- patients (n = 192). Patients with low disease activity (EDSS = 0.5–2.0; n = 65) showed significantly lower levels of LILRA3 when compared to patients with intermediate disease activity (EDSS = 2.5–5.5; n = 65; p<0.05) and to patients with high disease activity (EDSS = 6.0–8.0; n = 62; p<0.001) (Fig 5A). Consistent with this, there was significant positive correlation between increasing concentrations of serum LILRA3 and higher disease severity scores (Spearman r = 0.29, p = 0.002; n = 192) (Fig 5B). Multiple regression analysis showed that LILRA3 protein level, age of disease onset were highly significant independent indicators of disease severity as determined by EDSS (Table 4). In brief, one standard deviation (SD) increase in LILRA3 protein level predicted 0.19 SD increase in disease severity (β = 0.19, p = 0.006) and 1 SD increase in the median age of disease onset was linked to a 0.43 SD decrease in disease severity (β = -0.43, p = 0.001) (i.e. higher LILRA3 levels or younger age of disease onset were linked to more severe disease). Age at the time of blood collection was also positively associated with more severe disease, although less significant (Table 4). By contrast, sex or recent disease recurrence did not significantly contribute to EDSS (Table 4).

**Table 4:** Multiple regression analysis to evaluate the independent contribution LILRA3 to EDSS in context of other relevant covariates (n = 440).

Variable*	*P value*	*Standardised Coefficient (βeta)*
***LILRA3_log***	*0*.*006*	*0*.*192*
***Age of disease onset***	*0*.*001*	*-0*.*434*
***Sex***	*0*.*238*	*-0*.*081*
***Age at the time of blood collection***	*0*.*01*	*0*.*539*
***Recent disease recurrence***	*0*.*250*	*-0*.*079*

*Dependent variable: EDSS at the time of sera collection

### Associations between alteration in serum LILRA3 levels and changes in EDSS over time

**Fig 6:**
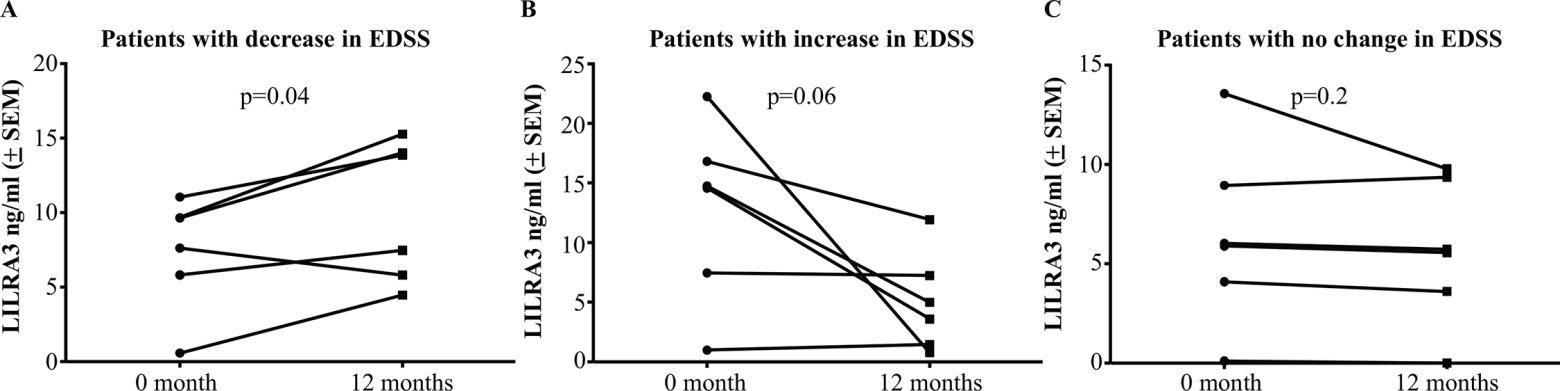
Association between altered serum LILRA3 levels and fluctuated EDSS over time. **(A)** Patients with RRMS that showed clinical improvement (decreased in EDSS score) at a 12 month follow-up had marked increase in LILRA3 levels in serum when compared to levels measured 12 months earlier (n = 6, paired t test, p = 0.04). **(B)** By contrast, patients that had worsening disease (increased in EDSS score) had markedly lower serum LILRA3 at the 12 month follow-up (n = 6, p = 0.06). **(C)** LILRA3 level remained steady in most patients with stable clinical scores (no change in EDSS score) (n = 6, p = 0.2).

To investigate whether LILRA3 levels change with fluctuations in clinical status, LILRA3 levels were measured at two time points (12 months apart) in 18 patients with RRMS who had sequential EDSS scores and corresponding serum samples. In patients showing clinical improvement, there was a significant increase in LILRA3 levels during the 12 month follow-up (n = 6, p = 0.04) (Fig 6A). By contrast, patients with worsening clinical disease had markedly lower serum LILRA3 during the 12 month follow-up compared with initial levels (n = 6, p = 0.06) (Fig 6B). There was no change in serum LILRA3 levels in patients with stable clinical scores (n = 6, p = 0.2) (Fig 6C).

### Correlations between LILRA3 and serum IL-10, IFNγ and TNFα levels in patients with MS

**Fig 7:**
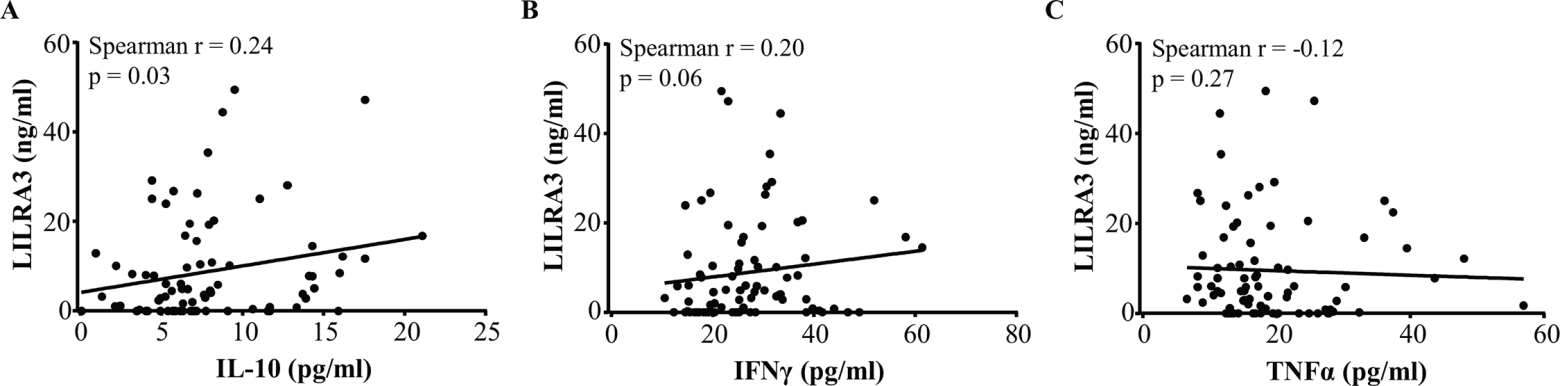
Correlations between LILRA3 and serum IL-10, IFNγ and TNFα levels in 80 patients with MS (A) Serum IL-10 concentrations in patients with MS showed significant positive correlation to LILRA3 levels in the corresponding sera (Spearman r = 0.24; p = 0.03). (B) Similarly, serum IFNγ in the same patients positively correlated to their LILRA3 levels but this was not statistically significant (Spearman r = 0.20; p = 0.06). (C) By contrast, there was negative association between serum TNFα and LILRA3, although the correlation was weak and did not reach statistical significance (Spearman r = -0.12; p = 0.27).

LILRA3 is upregulated in monocytes treated with recombinant IL-10 or IFNγ *in vitro* but not TNFα [31]and in psoriatic patients injected with IL-10 *in vivo* [32]. Thus, we proposed that IL-10, IFNγ and/or TNFα levels in sera of patients may regulate LILRA3 expression. To address this, serum IL-10, IFNγ and/or TNFα in 80 randomly selected patients was measured and levels correlated to the corresponding serum LILRA3. Serum LILRA3 levels positively correlated with IL-10 (Spearman r = 0.24) and this was statistically significant (p = 0.03) (Fig 7A). Positive correlation was also found with IFNγ (Spearman r = 0.20) but this was not statistically significant (p = 0.06) (Fig 7B). There was statistically non-significant (p = 0.27) weak negative correlation between TNFα and LILRA3 (Spearman r = -0.12) (Fig 7C), as opposed to the positive correlation observed with IL-10 and IFNγ.

## Discussion

A LILRA3 gene deletion polymorphism has been reported to be associated with several autoimmune diseases (reviewed by [28]). Our study found no association between LILRA3 deletion and the susceptibility of MS in a North American cohort. Genotypic and allelic frequencies of LILRA3 gene deletion in control subjects were measurably but not significantly higher than in patients. These findings are contrary to previous studies that reported associations between LILRA3 gene deletion and MS susceptibility in German [29] and Spanish [24] populations, but consistent with Polish [25] and Finnish [8] studies, which also did not find a link between LILRA3 deletion and MS susceptibility. Differences in genetic background among the different populations and sample sizes may partially explain the presence or lack of contribution of LILRA3 deletion to MS susceptibility. However, recent multicentre genome-wide association studies that included over 80,000 individuals of European ancestry also failed to reveal LILRA3 gene polymorphism as one of the more than 110 MS risk variants identified [10–13]. It is also important to note that the difference in the distribution of homozygous LILRA3 deletion between patients and controls in the German study was very small (3.3%), despite reaching statistical significance due to a large sample size [29]. Similarly, the results in one of the two Spanish cohorts (Hospital del Mar, Barcelona) was not significant and required pooling of two cohorts to achieve statistically significant effects [24]. Interestingly, a recent meta-analysis in another Spanish group indicated no link between LILRA3 gene deletion and MS risk [33]. Our own results do not support an association between LILRA3 gene deletion polymorphism and MS susceptibility. Moreover there was no link with clinical severity, clinical subtype or age of disease onset. Owing to these conflicting genetic study results, we proposed that measurement of serum LILRA3 protein may provide a clearer representation with regards to its association with MS.

We showed for the first time that serum LILRA3 protein was significantly upregulated in patients with MS compared to the healthy controls and, importantly increasing amount of LILRA3 in patient sera positively correlated with worsening disease severity. These results are similar to previous findings in rheumatoid arthritis that showed increasing amounts of LILRA3 with increasing disease activity scores [31], indicating that LILRA3 may play a role in the pathogenesis of diseases characterised by excessive unregulated chronic inflammation. Limited *in vitro* evidence shows that LILRA3 is a potent inhibitor of LPS-mediated TNFα production [34], and *in vivo* clinical association studies demonstrated that LILRA3 protein/mRNA are significantly up-regulated during inflammation [31, 35]. Moreover, LILRA3 gene and/or protein is significantly increased in response to *in vivo* and *in vitro* treatment of monocytes with the anti-inflammatory cytokine, IL-10 [31, 32], but not in response to *in vitro* treatment with the pro-inflammatory mediator, TNFα [31]. Here we showed significant positive correlation between the serum level of LILRA3 and one of the most potent anti-inflammatory cytokines, IL-10 but negative correlation to the protypical pro-inflammatory cytokine TNFα, albeit weakly. These new *in vivo* results are consistent with our previous *in vitro* observation [31] and may represent a new IL-10-LILRA3 counterregulatory feedback mechanism that downregulates inflammation in MS. LILRA3 is soluble and does not have membrane-bound counterpart, but its “ligand binding” extracellular domains are highly homologous to some activating LILRs [20], thus it may exert anti-inflammatory properties by acting as a soluble antagonist to these activating receptors, analogous to the soluble TNFα and IL-1 receptors [36]. In agreement to this proposal, we found substantial increase in LILRA3 levels in patients that showed clinical improvement over a 12 month period contrasted with patients that had worsening clinical scores had substantial decrease in LILRA3 levels. Similar expression patterns have been shown in some key molecules that are known to improve disease outcome in MS [37–40]. Particularly, elevated IL-10 was associated with disease remission in MS [37–40], and higher blood levels of vitamin D correlated with better clinical outcomes [41]. Moreover, it has been shown that patients that responded to treatment with IFNβ had substantially increased serum IL-10 [42–44], and vitamin D is proposed to modulate inflammation through up-regulation of IL-10 [45, 46]. It is intriguing whether the anti-inflammatory effects of IL-10 in this setting might be explained by its ability to induce mediators such as LILRA3 [31, 32] and failure to respond to IFNβ treatment in a subset of patients with MS might in part be due to deficiencies in secondary messengers such as LILRA3. Interestingly, we found elevated LILRA3 levels at 12 months follow up that were higher than the initial levels in a subset of patients despite their clinical improvement. This is likely due to ongoing subclinical inflammation and/or that the LILRA3 protein that was highly elevated in response to the initial disease reactivation may have persisted in circulation beyond the 12 months follow up due to long half life of the protein. Moreover, these patient might have continued with treatments that are known to directly or indirectly increase LILRA3 including IFNβ (unpublished, [43]) or vitamin D [45, 46]. It is also noteworthy that this study was performed in small number of patients requiring cautious interpretation of the data and warranting further investigations.

Analysis in patients with the different clinical subtypes showed that patients with primary progressive disease (PPMS) had the highest amounts of serum LILRA3 followed by patients with SPMS that had intermediate amounts and patients with RRMS that had the least amount. These results are in good agreement with our finding showing positive correlation between disease severities and increasing amounts of LILRA3, given that PPMS patients typically have more severe disease. Importantly, multiple regression analysis showed that serum LILRA3 level is one of the strongest positive predictors of disease severity in MS. These results suggest that serum LILRA3 might potentially be used as a biomarker for disease severity and/or as an additional objective indicator of clinical subtype in MS. Interestingly, here we confirmed that homozygous 6.7kbp LILRA3 gene deletion results in undetectable amounts of LILRA3 protein in sera. This is the first experimental confirmation of the predicted lack of LILRA3 translation in healthy subjects with homozygous LILRA3 gene deletion. The physiological significance of the absence of LILRA3 protein in a subset of healthy subjects remains to be elucidated.

Taken together our results indicate that LILRA3 is not a risk variant in MS susceptibility but is involved in modulating inflammation thereby contributing to the variability in disease severity and/or clinical outcomes. The exact functions of LILRA3 in MS require further investigation.
